# Enhanced Structural, Optical Properties and Antibacterial Activity of PEO/CMC Doped TiO_2_ NPs for Food Packaging Applications

**DOI:** 10.3390/polym15020384

**Published:** 2023-01-11

**Authors:** Ibrahim A. Alhagri, Talal F. Qahtan, Mohammed O. Farea, Ahmed N. Al-Hakimi, Sadeq M. Al-Hazmy, Saeed El-Sayed Saeed, Abuzar EAE Albadri

**Affiliations:** 1 Department of Chemistry, College of Sciences, Qassim University, Buraidah 51452, Saudi Arabia; 2 Department of Chemistry, Faculty of Sciences, Ibb University, Ibb 70270, Yemen; 3 Physics Department, College of Science and Humanities in Al-Kharj, Prince Sattam Bin Abdulaziz University, Al-Kharj 11942, Saudi Arabia; 4 Physics Department, Faculty of Science, Ibb University, Ibb 70270, Yemen

**Keywords:** PEO, CMC, TiO_2_, FTIR, TEM, AC conductivity

## Abstract

In this article, the synthesis, optical, and electrical properties of composites consisting of polyethylene oxide (PEO), carboxymethyl cellulose (CMC), and titanium dioxide nanoparticles are examined. Flexible nanocomposite samples comprising PEO, CMC, and TiO_2_ nanoparticles were produced swiftly via using the cast synthesis method. In addition, XRD and FT-IR analysis were performed in order to analyze the structures of the prepared samples. Our results demonstrate the PEO/CMC blend’s effectiveness in interacting with TiO_2_ nanoparticles. The optical properties of the PEO/CMC and nanocomposite samples, such as the energy band gap, were studied using the UV/Vis optical absorbance. It was found that as TiO_2_ NP weight fraction increases, the energy gap narrows. Moreover, TiO_2_ nanoparticles with an average size of 16 nm were formed in spherical and rod shapes, according to a TEM image. The SEM images demonstrate how the distribution of TiO_2_ NPs increased upon the surfaces of the prepared films. The antibacterial activity in the nanocomposites was shown to be enhanced by the TiO_2_ NP concentrations. Finally, we proposed that PEO/CMC-0.8 wt. % TiO_2_ nanocomposites with enhanced optical, electrical, and dielectric properties should be used in electrochemical devices.

## 1. Introduction

Polymer blending has developed into a significant and convenient method for the purposes of creating novel material with altered physical properties in comparison to pure polymer [1]. In addition to possessing high thermal stability, semicrystalline-linear polyethylene oxide (PEO) is useful for supporting mechanical properties and also possesses a low cost. Therefore, PEO are being considered in respect to using them in antibacterial coatings, as well as in the food packaging industry [2,3]. As an effective coating technique, the PEO polymer has been used to produce antimicrobial coatings on the surfaces of bio-medical devices. Although PEO coating alone can enhance the antimicrobial activity of materials, additions of nanofiller with good antibacterial activity into the PEO matrix can further double the antimicrobial activity [4,5]. Since carboxymethyl cellulose (CMC) polymer is amorphous, it facilitates the movement of ions within the blend matrix by increasing the number of amorphous regions. Having said this, to the best of our knowledge, PEO/CMC has only been studied to small degree in the literature [6,7,8]. CMC is one of the more important cellulose derivatives due to the fact that it is an anionic water soluble polymer. Additionally, it provides certain valuable properties, including filming, suspension, emulsification, water maintenance, biodegradability, and nontoxicity. It has different uses—for example in food packaging, bathroom products, and paper manufacturing [9]. Indeed, the unique properties and endless potential of nanoparticles have fascinated those involved in materials science [10]. The large surface area of nanoparticles increases their reactivity [11]. Consequently, nanoparticles formed in an organic polymeric matrix have the potential to create a range of practical devices, such as optoelectronics, memory, solar cells, and energy storage systems [12]. Due to its controlled properties in a variety of applications—including integrated capacitors, intelligent structures, actuators, and acoustic emission sensors—nanoscale polymers containing electroactive ceramics have gained considerable interest [13]. The photocatalytic process uses of TiO_2_ nanoparticles are an easy and promising method to prevent bacterial infection. In the crystal structure of TiO_2_, each Ti^4+^ ion is surrounded by an octahedron of six O^2−^ ions, while each oxygen atom is surrounded by three Ti atoms. TiO_2_ can also be used as the most photoactive phase and is a widely applicable semiconductor material for the purposes of environmental application. On the other hand, piezoelectric transducers and other uses rely on their ferroelectric properties [14,15]. Many scientists have looked into the effects of TiO_2_ incorporation in various polymeric matrices [16,17,18]. For example, L.H. Gaabour [16] investigated the impact of adding TiO_2_ NPs, which were created using the sol-gel method, to a blend of PS and polyvinyl chloride (PVC) polymers with a ratio of 50:50 wt. % by the casting process. He discovered that adding TiO_2_ to the (PEO)6: NaPO_3_ matrix at varying ratios improved its structural and electrical properties. Abutalib and Rajeh [18] synthesized TiO_2_ nanoparticles via the sol-gel method. In addition, the TEM micrograph showed that the size of the TiO_2_ nanoparticles was visible at the nanoscale. The TiO_2_ nanoparticles were added to the SA/PANi blend to obtain the nanocomposite samples. They observed that the filled samples showed excellent antibacterial activity against Gram-positive and Gram-negative bacteria. The highest antibacterial activity of the nanocomposite samples were found against *S. aureus* and *E. coli* bacteria. Additionally, Jayanthi et al. [19] examined the influence of nanoscale TiO_2_ on the morphological, electrical, and optical properties of PEO-PVC-LiClO_4_ electrolytes. We are aware of no prior studies on the creation of the PEO/CMC composite while incorporated with TiO_2_ NPs. The purpose of this research is to use X-ray diffraction, FT-IR, and UV/Vis. spectroscopy to investigate the effect of TiO_2_ packing on the structural, optical, and antibacterial activity of the PEO/CMC composite.

## 2. Experimental

### 2.1. Chemicals

ACROS Organic in Morris Plains, New Jersey provided the PEO polymer, which has an MW = 35,000 g·mol^−1^, while BDH Chemical Ltd. provided the CMC, which has an MW = 190,000 g·mol^−1^ (Poole, UK). The TiO_2_ was supplied by Sigma-Aldrich (Schnelldorf, Germany) and had a purity of 99.997%. During the examination, the chemicals were dissolved in double distilled water (DDW) obtained from the Al-gomhoria company located in Mansoura, Egypt.

### 2.2. Creation of PEO/CMC-TiO_2_ Nanocomposite

In double distilled water, CMC and PEO were separately dissolved at the required concentrations. The solution became crystal clear and transparent after four hours. The prepared samples comprised around 30% PEO and 70% CMC. Various weight percentages of the polymer nanocomposites (i.e., 0.0, 0.10, 0.20, 0.40, and 0.80 wt. %) were obtained after dispersing TiO_2_ for five hours in double-distilled water. Subsequently, a homogeneous viscous liquid was produced by combining all the solutions and vigorously agitating them. The prepared solutions were cast into a Petri dish. When the solvent gradually evaporated for two days, polymeric mixtures were modified into nanocomposites. The nanocomposites of PEO/CMC-TiO_2_ were then peeled from the Petri dish and used for further studies.

### 2.3. Characterization

A XRD diffraction study of obtained films with varying TiO_2_ concentrations was performed using DIANO XRD 800 diffractometers with a Ni filter Cu Kα radiation of λ = 1.54 nm. The functional groups in respect of the PEO/CMC-TiO_2_ nanocomposites were determined using FTIR spectroscopy (Nicolet iS10, Minneapolis, MN, USA). The UV-Vis spectra of polymer nanocomposite films were measured with an RT utilizing a JASCO UV-VIS spectrophotometer (Model V-630, Japan). The size and shape of the gold nanoparticles were investigated using a TEM (JEOL–JEM–1011, Tokyo, Japan). The produced samples’ surfaces were examined using scanning electron microscopy (JEOLJSM –6510 LV, Peabody, MA, USA).

## 3. Results and Discussion

### 3.1. XRD Spectra

**Figure 1:**
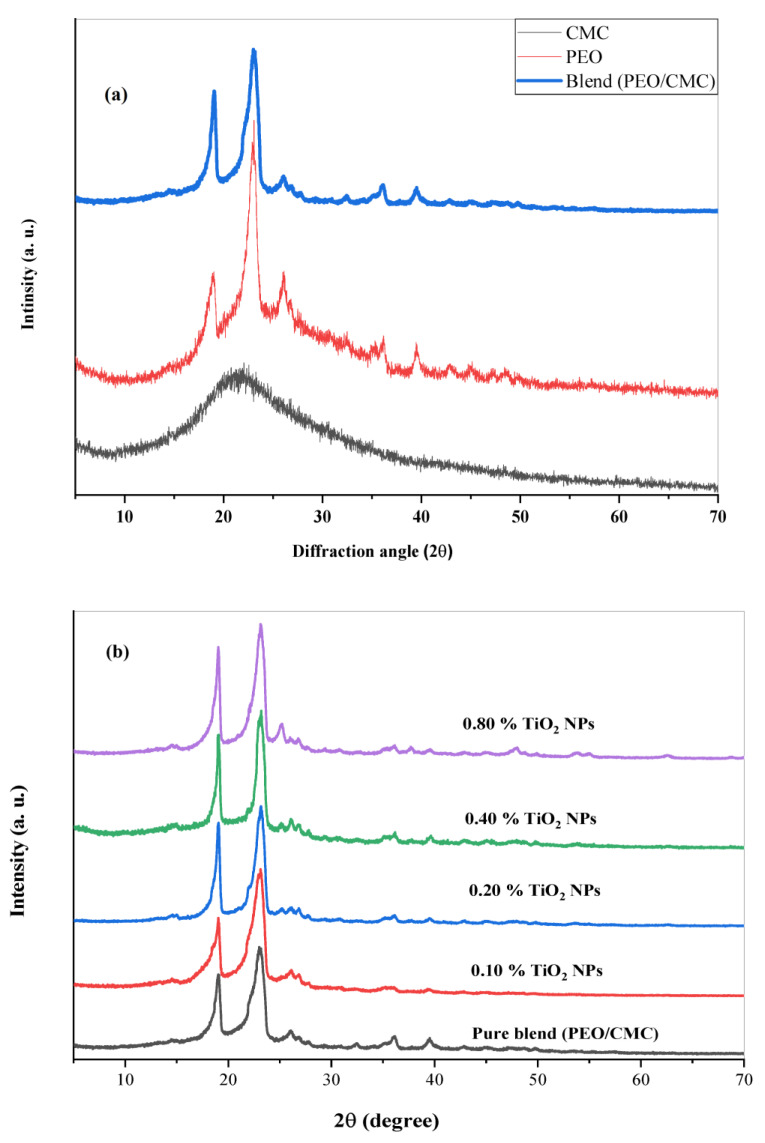
XRD scans of (**a**) pure PEO, pure CMC, and PEO/CMC composite. (**b**) PEO/CMC filled with different amounts of TiO_2_ NPs.

Figure 1a displays the XRD spectra for PEO, CMC, and the PEO/CMC composite. The PEO spectrum contains three major sharp peaks at 2θ = 18.85°, 22.82°, and 26.07, thereby demonstrating the semi-crystalline nature of PEO. These three peaks are connected by plane (112), (120), and (121) [PCPDF File. No, 49-2234 and 49-2200] [20]. The amorphous character of CMC is indicated by the presence of a broad peak in its spectral distribution at 2θ = 21.16°. An XRD pattern for the PEO/CMC composite and its nanocomposites containing TiO_2_ nanoparticles is shown in Figure 1b. The semicrystalline character of the composite can be seen in the XRD spectra, which feature two primary strong peaks at 19.04° and 23.06°, due to the presence of the semicrystalline polymer, PEO. The composite sample’s XRD spectrum has many low-intensity peaks at 2θ = 27.07°, 32.44°, 36.06°, and 39.57°. The two main diffraction peaks of the nanocomposite films show a reduction in intensity and an expansion in width. Furthermore, when filler concentrations increase, the intensity of the peaks at 2θ = 19.04°, 23.06°, and 26.07° increases. The distribution of TiO_2_ within the PEO/CMC chain becomes random as a result of these observations. Indeed, these are supported by FTIR measurements, which show a strong interaction between TiO_2_ and PEO/CMC [21]. Due to this interaction, the nanocomposite’s degree of crystallinity falls and its proportion of amorphous regions rises, thereby enhancing the conductivity of the PEO/CMC/TiO_2_ nanocomposite. The XRD patterns for the nanocomposite films do not show any new peaks. This demonstrates that the TiO_2_ NPs within the PEO/CMC matrix have completely dissolved.

### 3.2. FTIR Investigation

**Figure 2:**
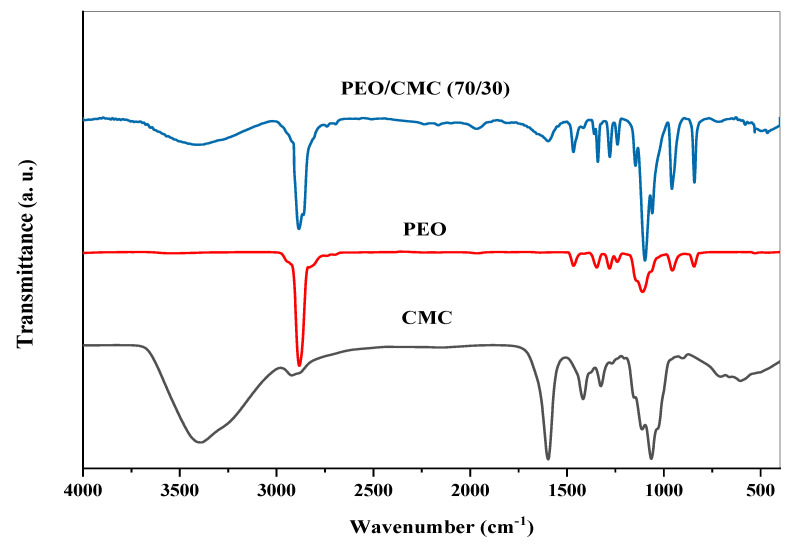
FTIR spectra of pure PEO, CMC, and the pure (PEO/CMC) blend.

**Figure 3:**
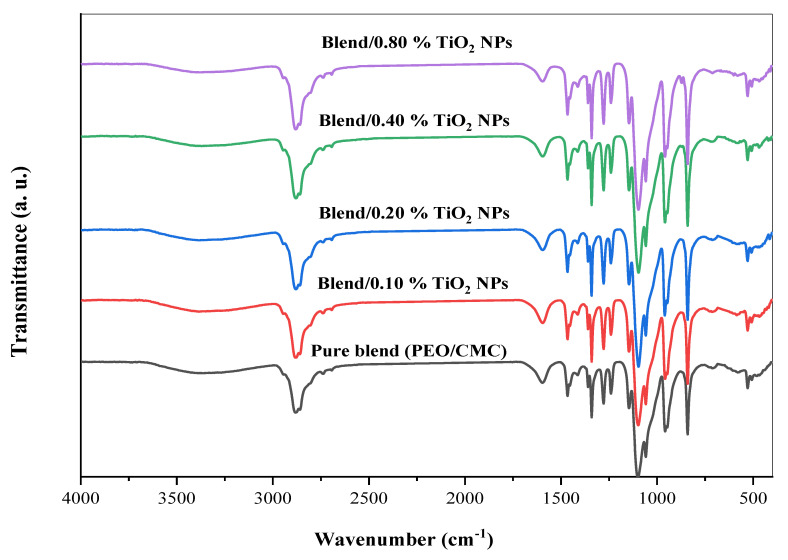
FT-IR transmittance spectra of PEO/CMC/TiO_2_ NP nanocomposite samples.

It is possible to study intermolecular interactions in self-polymers or between several polymers in a polymeric matrix by using FTIR spectroscopy. The interaction between TiO_2_ nanoparticles and the PEO/CMC composite was studied using FTIR spectroscopy in this article. Figure 2 displays illustrations of the FTIR analysis of CMC, PEO, and the PEO/CMC blend. Figure 3 displays illustrations of the FT-IR analysis of PEO/CMC, as well as the PEO/CMC filled with 0.0, 0.1, 0.2, 0.4, and 0.8 wt. % concentrations of TiO_2_ NPs. The existence of titanium dioxide affects a broadening of the hydroxyl group, thereby stretching the vibration to 3454 cm^−1^. The peak of the CH_2_ asymmetric stretching of PEO is detected at 2884 cm^−1^; further, its broadness is somewhat changed. The stretching vibration of C=O of CMC was thought to be responsible for the peak at 1600 cm^−1^. After CMC was bended with PEO, there was a decrease in the transmittance of this band (1600 cm^−1^) as observed in Figure 2. The band at 1475 cm^−1^ corresponds to the scissoring of CH_2_ in respect of PEO. The asymmetric bending of CH_2_ is responsible for a transmittance band for PEO at 1334 cm^−1^. By increasing filler concentration, the small transmittance band at 1240 cm^−1^, caused by a symmetric CH_2_ twisting of PEO, remains unchanged. With increasing filler concentration, the transmittance band at 1090 cm^−1^ increases substantially, indicating a stretching of the C–O–C group within the PEO chain. There were two main transmittance bands at 958 and 817 cm^−1^. The CH bending mode and C–C stretching created these peaks; in addition, these bands were typical of PEO polymers. As a result of these findings, PEO, CMC, and the PEO/CMC blend with TiO_2_ have strong vibrational interactions between their vibrational groups. Table 1 lists the FTIR characterization band assignments for (a) PEO, (b) CMC, and (c) the PEO/CMC blend. When different concentrations of TiO_2_ NPs are added to the PEO/CMC samples, the interaction between organic and inorganic components affects the vibrational bands of the polymeric chain. The different transmittance values for the CH_2_, C=O, C–O–C, and OH groups serve as an example of this interaction. The transmittance in the vibrational groups C–O, C=O, and CH_2_ were dramatically altered, indicating a powerful interaction between the polymer matrix and TiO_2_ NPs. An X-ray diffractometry (XRD) examination that showed the breakdown of the polymer matrix’s crystal structure confirmed these results. This suggests that the PEO/CMC and TiO_2_ NP vibrational groups are intricate and interacting.

### 3.3. UV-Vis Spectroscopy

**Figure 4:**
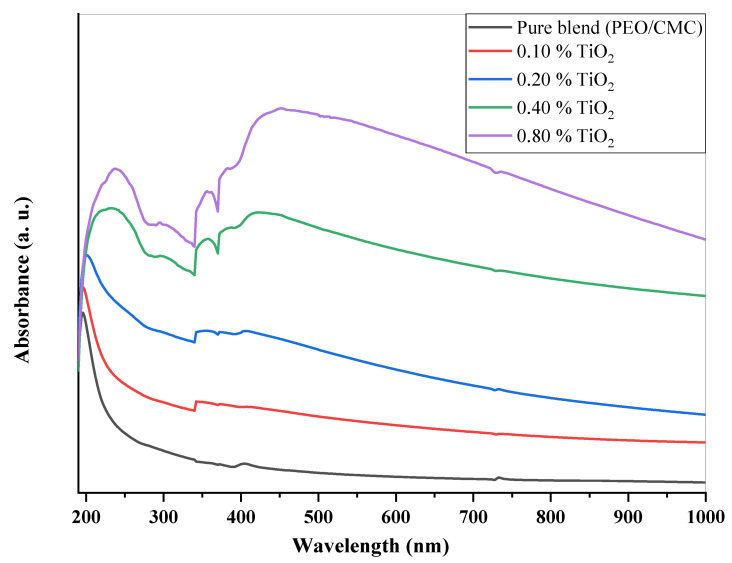
UV/vis spectra of PEO/CMC/TiO_2_ NP nanocomposites.

**Figure 5:**
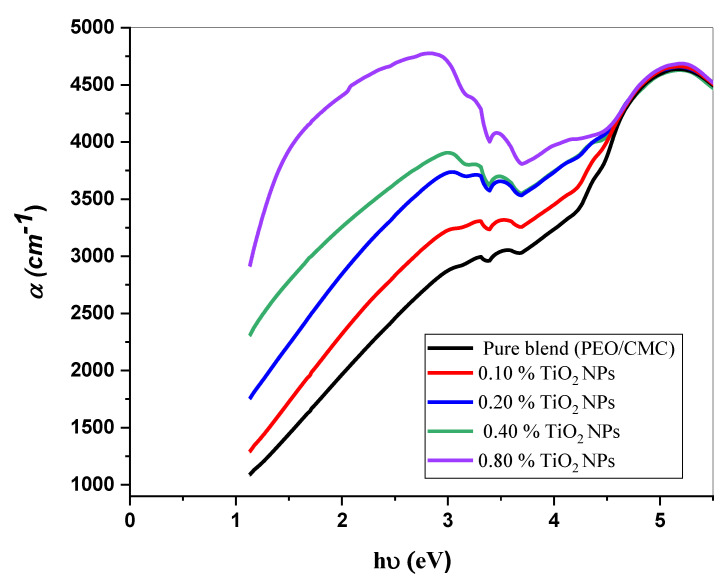
The relation between α(v) and *h*v for the PEO/CMC blend and PEO/CMC/TiO_2_ NPs at various concentrations.

**Figure 6:**
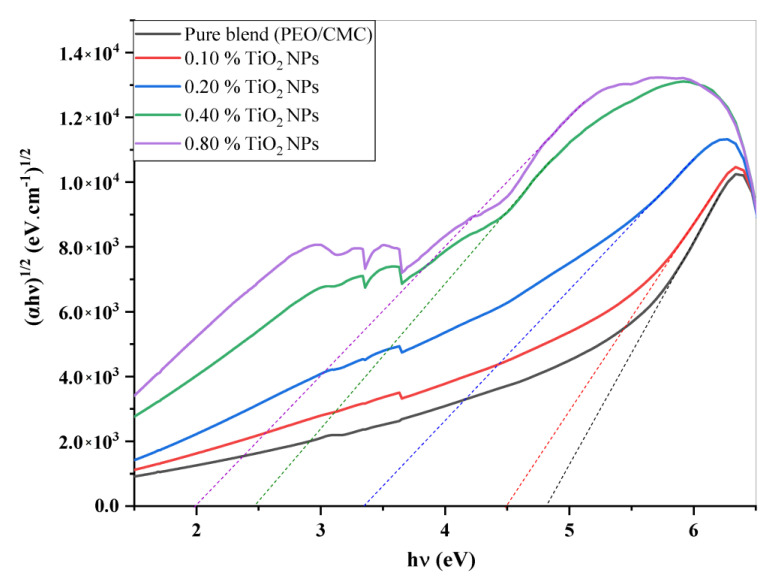
Plots of (*αhυ*)^1/2^ vs. *hυ* of PEO/CMC and PEO/CMC filled with various amounts of TiO_2_ NPs.

**Figure 7:**
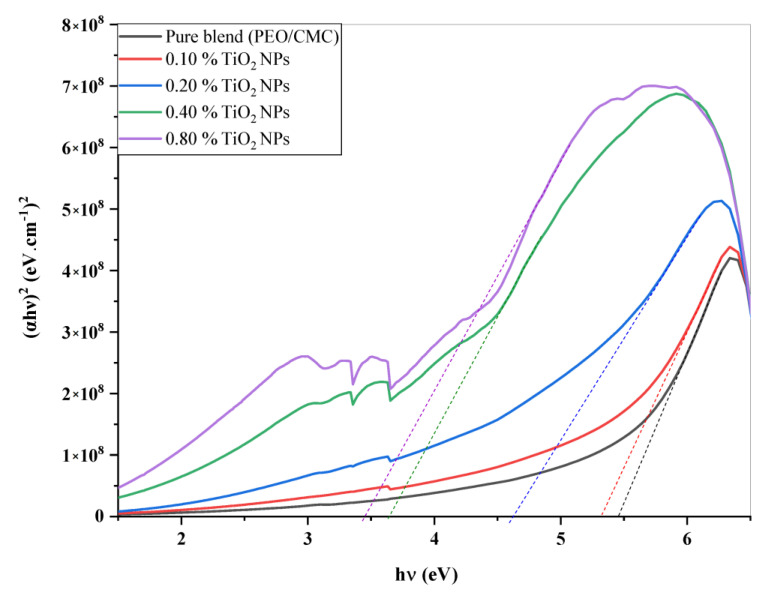
Plots of (α*hυ*)^2^ vs. *hυ* of the PEO/CMC blend and the PEO/CMC filled with various amounts of TiO_2_ NPs.

UV/Vis analysis is the process of determining how much a light beam attenuates after passing through a sample or after reflecting off of a sample surface. Figure 4 shows the ultraviolet and visible absorption spectra for the PEO/CMC blend and PEO/CMC-TiO_2_ NPs at different concentrations. The shoulder peak in the PEO/CMC spectrum at 196 nm is caused by an n→ π* transition. Another peak, belonging to π → π*, is located at 225 nm [22]. The 225 nm peak’s strength increases and is shifted to longer wavelengths when the concentration of TiO2 increases (redshift). As determined by XRD analysis, the shift of the TiO_2_ NPs could be due to changes in crystallinity, which suggest complexation/homogeneity, and differences in the energy gaps between polymeric matrices and TiO_2_ NPs [4,21,23,24]. The last two samples (at 0.4 and 0.8 wt. %) show a new peak at 414 nm, which could be due to titanium nanoparticle surface plasmon resonance (SPR). It appears that the PEO/CMC matrix contains titanium nanoparticles, as evidenced by the presence of an SPR peak. There is an increase in surface plasmon resonance intensity and a shift toward longer wavelengths (redshift from 414 to 444 nm). A combination of PEO/CMC and TiO_2_ nanoparticles was modified in order to confirm their interaction and complexation. The energy gap is calculated using the following equation (*E_g_*) [15,25]:

(1) $$\left(\alpha hv\right)=C{\left(hv-E_{g}\right)}^{r}$$

where *h*v denotes the photon energy and *C* denotes constant. The *r* value varies depending on whether an electronic transition is indirect or direct. In addition, it has values of 2 and ½ in the k space, respectively. The Beer–Lambert formula can be utilized to determine an absorption coefficient α [22].

(2) $$\alpha \left(v\right)=2.303\left(\;\frac{A}{d}\;\right)$$

where *A* denotes the absorbance and *d* indicates the thickness of the investigated films. Figure 5 shows the relation between α(v) and *hυ* for the PEO/CMC blend and the PEO/CMC/TiO_2_ NPs. Table 2 shows the absorption edge values, which have decreased from 2.43 eV to 2.13 eV. The absorption edge values decrease as filler concentration increases, due to the electron hole’s conduction and valence bands changing. Figure 6 and Figure 7 depict the plots of (αhυ)^1/2^ and (α*hυ*)^2^ versus *hυ*; Table 2 contains a listing of the values that were obtained. As shown in Table 2, the energy gap value, *E_g_*, decreases as filler concentrations rise from 5.46 eV–3.45 eV for direct transition and 4.81 eV–1.99 eV for indirect transition. This decrease may be caused by the coordination or interaction between the TiO_2_ NPs and PEO/CMC matrix, which may lead to localized states inside the band gap.

### 3.4. Analysis of HR-TEM Image

**Figure 8:**
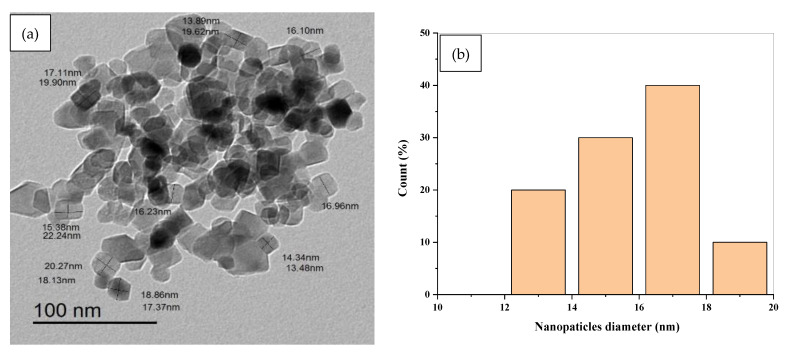

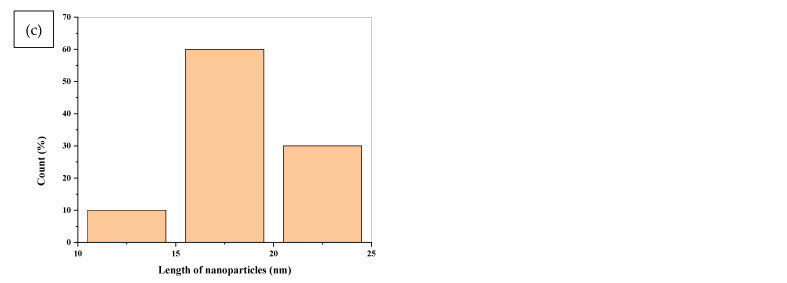
(**a**) TEM image of TiO_2_ NPs (**b**) the corresponding histograms of diameter TiO_2_ NPs (**c**) the corresponding histograms of length distribution of TiO_2_ NPs.

The particle size and crystallinity of samples were examined further using HR-TEM images. Figure 8a–c shows an HRTEM image together with the accompanying histograms for the diameter and length distributions of TiO_2_ nanopowder in the anatase phases. The grain size of the particles, which was less than 20 nm, indicates that they are nanoscale in size. The TiO_2_ nanopowder in the anatase phase consists of both spherical and rod particles with an average length of 17.5 nm, as revealed in histogram (c). Moreover, it may be deduced that the sample’s particles are nanoscale in size, with grain sizes ranging between 12 and 20 nm.

### 3.5. FE-SEM Images

**Figure 9:**
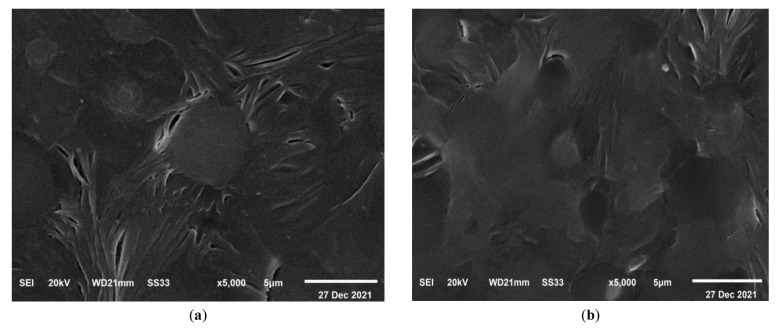

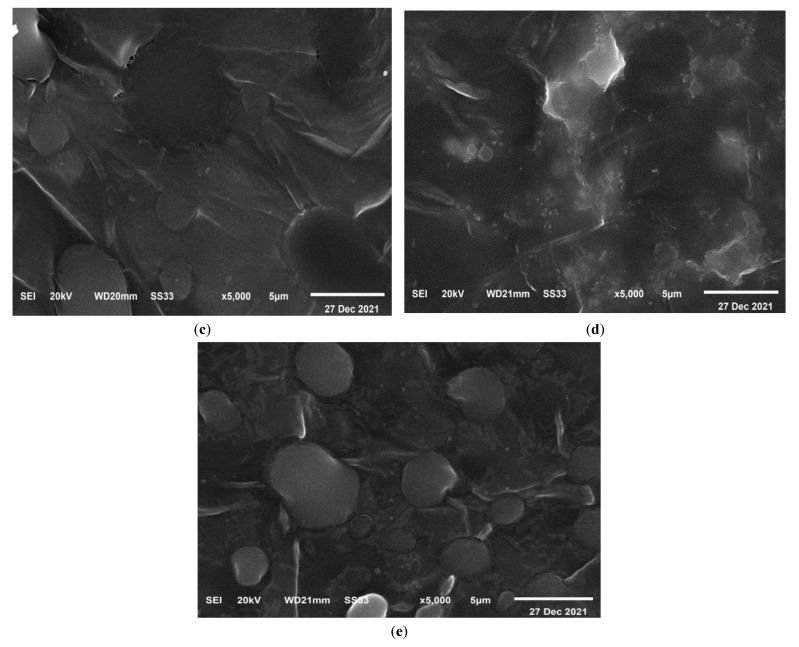
SEM images of (**a**) the pure PEO/CMC blend, (**b**) 0.10, (**c**) 0.20, (**d**) 0.40, and (**e**) 0.80 (wt. %) of TiO_2_ NPs at mag. of 5000 times.

The characteristic SEM micrographs of the prepared films that are under investigation, were received at a magnification of 5000 times and are shown in Figure 9. This micrograph represents a blend of PEO and CMC. The surface is smooth and has spherical pores distributed regularly, as seen in Figure 9a. This pore structure is partially filled by TiO_2_ when it is added to a polymer blend as shown in Figure 9b–e. When compared to the pure samples (PEO/CMC), the pores are smaller for the TiO_2_ content of 0.8%, and the surface is coarser for the 0.8% film due to the fact that most of the pores have been filled with nanoparticles. As a result, incorporating TiO_2_ into the PEO/CMC blend has significantly affected surface morphology, which can be described as the physical interaction between metal oxides and organic materials. It is interesting to observe that there was no evidence of phase separation in these micrographs. This gives a clear indication regarding how well the PEO/CMC mixture interacts with TiO_2_ nanoparticles, demonstrating that their complexation was acceptable.

### 3.6. Antibacterial Assay

**Figure 10:**
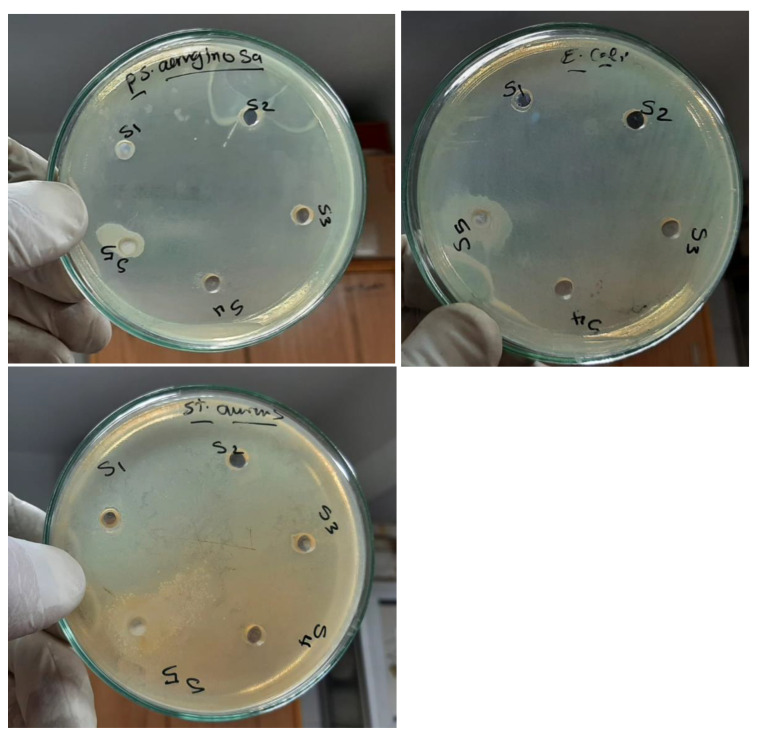
Antibacterial inhibition zones of the PEO/CMC composite with a differing content of TiO_2_ NPs: (S_1_) PEO/CMC, (S_2_) 0.10, (S_3_) 0.20, (S_4_) 0.40, and (S_5_) 0.80 wt. % TiO_2_ NPs.

Figure 10 exhibits the in vitro activity index assay of the obtained films versus three types of bacteria: Pseudomonas aeruginosa, Escherichia coli, and Staphylococcus aureus as controls. An antibacterial test was recorded after 24 h of incubation under UV irradiation in order to record the inhibition zone diameters. The findings exhibited that as the amount of TiO_2_ NPs increased, antibacterial activity also steadily increases. In the examination, antimicrobial broad-spectrum actions are clearly discernible. The findings indicated that raising the concentration of TiO2 NPs increased activity against the studied bacterial species, but that Pseudomonas aeruginosa had the greatest impact on the antibacterial assay [23,24]. The species from the samples were found on the agar plates that had been sown with the test organisms. At 37 °C, dishes were incubated. Table 3 lists the inhibition area sizes (mm) for the microorganisms that were noticed after 24 h. These results showed how the pure PEO/CMC blend and its nanocomposites affected the inhibition area and activity index [26]. The diameter of the areas and the activity index are observed to rise in all films when the concentration of TiO_2_ NPs increases. The findings showed that the *S. aureus* bacteria had higher diameter zones and activity indices than the *E. coli* and *P. aeruginosa* bacteria. The operation of the Ti ions release mechanism—which results in variations in permeability, the creation of reactive oxygen species, metabolic disruption, and finally death as a result of the bacterial cells being destroyed—was what caused the inhibition of the examined region.

The growing of the antibacterial activity of TiO_2_ nanoparticles was due to their ability to activate free OH radicals and as per the H–O–O radicals by TiO_2_ nanoparticles. An important contribution to the biocidal process of TiO_2_ nanoparticles are found in cell membrane interaction due to their nanosize and penetrating ability. This is after which the TiO_2_ nanoparticles release reactive species through interaction with the inert environment of the cells. The produced radicals caused the destruction of bacterial cells due to their decomposition and complete degradation of microorganisms [27,28,29]. These findings demonstrated that PEO/CMC/TiO_2_ NPs films could serve as a viable substitute for their synthetic equivalents in the food packaging sector.

## 4. Conclusions

We swiftly formed flexible nanocomposite samples including TiO_2_ NPs as a nanofiller, CMC (carboxymethyl cellulose), and PEO (polyethylene oxide) via cast synthesis. In this article, the optical, electric properties, and antibacterial activity of composites made of polyethylene oxide were studied. In addition, the carboxymethyl cellulose was incorporated by the TiO_2_ nanoparticles. The disorder of the polymer chain was exacerbated after the TiO_2_ nanoparticles were embedded, according to X-ray diffraction data. The interaction between the bands corresponding to the OH, CH_3_, and CH_2_ vibrations modes, as well as to the TiO_2_ NPs were corroborated by the complexation between the PEO/CMC composite and the TiO_2_ NPs in the FTIR spectrum. The existence of a surface plasmon resonance peak at 414 nm for the titanium NPs is visible in the UV-visible analysis of PEO/CMC and TiO_2_ nanoparticles, which is the hallmark of titanium nanoparticles. An increase in TiO_2_ content results in the band gap decrease and antibacterial activities increase. The study of microstructure was performed in respect to HR-TEM and FE-SEM, in terms of proving the nanosized dimensions of inorganic particles and their homogeneous distribution in the bulk. Obtained materials exhibit synergetic properties in addition to the properties of interacting components. Increased antimicrobial efficacy was observed when the TiO_2_ NPs concentration was increased. As such, these results can be used in designing new materials for electronic devices.

## Figures and Tables

**Table 1:** The FTIR characterization peaks for PEO, CMC, and the PEO/CMC composite.

Wavenumber (cm^−1^) ^a^	Vibrational Modes ^a^
2884	CH_2_ asymmetric stretching
1465	CH_2_ scissoring
1348	CH_2_ asymmetric bending
1278	CH_2_ twisting
1236	CH_2_ symmetric twisting
1110	COC stretching
956	CH bending mode
844	CO stretching
**Wavenumber (cm^−1^) ^b^**	**Vibrational Modes ^b^**
3454	OH stretching
2956	CH_2_ asymmetric stretching
1600	stretching vibration of C=O
1427	bending vibration of C–H
1285	stretching vibration of C–N
844	C–C stretching
741	Out—of—plane rings CH bending
650	C—N bending
**Wavenumber (cm^−1^) ^c^**	**Vibrational Modes ^c^**
3454	OH stretching
2884	CH_2_ asymmetric stretching
1600	stretching vibration of C=O
1445	CH_2_ scissoring
1334	CH_2_ asymmetric bending
1240	stretching vibration of C–N
1090	COC stretching
958	CH bending mode
817	C–C stretching

a: The FTIR characterization peaks for PEO, b: The FTIR characterization peaks for CMC, c: The FTIR characterization peaks for the PEO/CMC composite.

**Table 2:** Optical energy gaps (*E_gi_* and *E_gd_*) and the absorption edge values for PEO/CMC/TiO_2_ NPs samples.

The Concentration of TiO_2_ NPs (wt. %)	Abs. Edge. (eV)	*E_g_* (eV)	
		Direct	Indirect
0.00	2.43	5.46	4.81
0.10	2.32	5.32	4.5
0.20	2.25	4.61	3.35
0.40	2.19	3.65	2.48
0.80	2.13	3.45	1.99

**Table 3:** Antibacterial activity of PEO/CMC filled with different content of TiO_2_ NPs.

Content of TiO_2_ NPs	*P. aeruginosa* Diameter of Area (mg/mL)	*E. coli* Diameter of Area (mg/mL)	*S. aureus* Diameter of Area (mg/mL)
0.00 wt. %	-ve	-ve	-ve
0.10 wt. %	2	3	6
0.20 wt. %	7	10	18
0.40 wt. %	11	13	33
0.80 wt. %	15	18	43

## Data Availability

The data that support the findings of this study are available on request from the corresponding author.
